# Transcriptional and Post-transcriptional Mechanisms Limit Heading Date 1 (Hd1) Function to Adapt Rice to High Latitudes

**DOI:** 10.1371/journal.pgen.1006530

**Published:** 2017-01-09

**Authors:** Daniela Goretti, Damiano Martignago, Martina Landini, Vittoria Brambilla, Jorge Gómez-Ariza, Nerina Gnesutta, Francesca Galbiati, Silvio Collani, Hiroki Takagi, Ryohei Terauchi, Roberto Mantovani, Fabio Fornara

**Affiliations:** 1 Department of Biosciences, University of Milan, Via Celoria 26, Milan, Italy; 2 Umeå Plant Science Centre, Department of Plant Physiology, Umeå University, Umeå, Sweden; 3 Department of Plant Biology and Crop Science, Rothamsted Research, Harpenden, United Kingdom; 4 Department of Agricultural and Environmental Sciences–Production, Territory, Agroenergy, University of Milan, Via Celoria 2, Milan, Italy; 5 Iwate Biotechnology Research Center and Laboratory of Crop Evolution, Graduate School of Agricultural Sciences, Kyoto University, Mozume, Muko, Kyoto, Japan; Chinese Academy of Agricultural Sciences, CHINA

## Abstract

Rice flowering is controlled by changes in the photoperiod that promote the transition to the reproductive phase as days become shorter. Natural genetic variation for flowering time has been largely documented and has been instrumental to define the genetics of the photoperiodic pathway, as well as providing valuable material for artificial selection of varieties better adapted to local environments. We mined genetic variation in a collection of rice varieties highly adapted to European regions and isolated distinct variants of the long day repressor *HEADING DATE 1* (*Hd1*) that perturb its expression or protein function. Specific variants allowed us to define novel features of the photoperiodic flowering pathway. We demonstrate that a histone fold domain scaffold formed by GRAIN YIELD, PLANT HEIGHT AND HEADING DATE 8 (Ghd8) and several NF-YC subunits can accommodate distinct proteins, including Hd1 and PSEUDO RESPONSE REGULATOR 37 (PRR37), and that the resulting OsNF-Y complex containing Hd1 can bind a specific sequence in the promoter of *HEADING DATE 3A* (*Hd3a*). Artificial selection has locally favored an Hd1 variant unable to assemble in such heterotrimeric complex. The causal polymorphism was defined as a single conserved lysine in the CCT domain of the Hd1 protein. Our results indicate how genetic variation can be stratified and explored at multiple levels, and how its description can contribute to the molecular understanding of basic developmental processes.

## Author Summary

Many plant species flower in response to changes in day length and can be categorized depending on their requirements for long or short days. Rice has tropical origins and normally flowers in response to shortening days. However, artificial selection operated by ancient farmers or modern breeders adapted rice cultivation to several environments, including those typical of temperate regions characterized by long days during the cropping season. Modifications of the genetic network controlling flowering that are causal to such expansion have been the subject of extensive studies, but the full complement of genes that regulate it and the molecular bases of their activity remains unknown. We took advantage of germplasm cultivated in Europe—and highly adapted to flower under long days–to isolate widespread variants of the *HEADING DATE 1* (*Hd1*) gene that limits flowering in temperate areas, and showed that such variants are non-functional and unable to prevent long day flowering. We identified the DNA changes causing the gene to be non-functional and used such mutant alleles as tools to demonstrate that Hd1 can bind a specific DNA sequence in the promoter of a florigenic rice gene. Mining genetic diversity becomes thus instrumental to define the molecular properties of regulatory pathways.

## Introduction

Rice (*Oryza sativa* L.) was originally domesticated ~8000 years ago in tropical Asia. Archeological remains and genome re-sequencing indicated southern China as the region of first cultivation, despite the debate regarding the domestication dynamics is still open [1–3]. Although of tropical origin, rice is currently cultivated across a broad latitudinal range [4,5]. Expansion to temperate areas required selection of varieties better adapted to local environmental conditions. Tolerance to low temperatures and day length-insensitive flowering have been crucial adaptive traits under selection.

Flowering (or heading date) is the result of an intricate series of pathways that mediate between environmental inputs and the production of molecules inducing flowering, known as florigens. Photoperiod is the major environmental cue that rice plants utilize to measure seasonal time [6]. Rice, as well as other important cereals including sorghum and maize, is a short day (SD) plant in which flowering is induced as the duration of the light phase during the day does not exceed a critical threshold. Under such inductive conditions, expression of *HEADING DATE 3a* (*Hd3a*) and *RICE FLOWERING LOCUS T1* (*RFT1*), florigenic genes highly similar to Arabidopsis *FLOWERING LOCUS T* (*FT*), is induced in the vascular tissue of leaves [7–11]. Expression of the florigens is triggered by *EARLY HEADING DATE 1* (*Ehd1*) encoding a B-type response regulator protein central to the photoperiodic flowering network [12]. The *Ehd1* gene is unique to rice and its transcription is strongly controlled at diurnal and seasonal levels [12,13]. Mapping of QTLs identified several major regulators of *Ehd1* expression that, upon cloning, were shown to encode transcription factors belonging to distinct protein groups [14–20]. In particular, *GRAIN YIELD*, *PLANT HEIGHT AND HEADING DATE 7* (*Ghd7*) and *PSEUDO RESPONSE REGULATOR 37* (*PRR37*) encode CCT (CONSTANS, CONSTANS-like, TOC1) domain proteins, whereas *Ghd8* encodes the NF-YB11 subunit of the NF-Y transcription factor complex [14–16]: all of them encode strong floral repressors. Artificial selection of rice varieties adapted to grow in Europe or Asia has taken advantage of loss-of-function alleles at such loci, because de-repression of *Ehd1* expression results in up-regulation of the florigens and subsequent flowering also under non-inductive day lengths [14–16,21,22]. Sensitivity to day length can be compromised to the extent that pyramiding of specific mutations completely abolishes it [23,24].

A major repressor of *Ehd1* transcription is encoded by *HEADING DATE 1* (*Hd1*), a zinc-finger CCT-domain transcription factor, homologous to *CONSTANS* of Arabidopsis [10,23,25,26]. As opposed to CO that promotes flowering under inductive long days (LD), Hd1 performs a dual function, because under LD conditions it delays flowering, whereas under SD conditions it promotes it by inducing expression of the florigens [10,27]. Similarly to floral repressor genes already mentioned, extensive allelic variation has been described at the *Hd1* locus that includes a plethora of loss-of-function alleles associated to varieties adapted to a broad latitudinal range [21,23,28–31]. However, the dual molecular function of Hd1 and the modes of repression of florigenic loci under LD are poorly understood. Although extensive genetic variation for flowering time traits has been described, more allelic variants must exist within local germplasm collections, because described genetic diversity appears insufficient to fully account for reduced sensitivity to day length of all varieties. Additionally, most efforts have focused on the identification of polymorphisms creating clear loss-of-function mutations, such as frame shifts or premature stops codons. Such first level of investigation provides an important but limited description of standing variation and additional levels have to be explored.

Comparisons between rice and Arabidopsis can help to derive and test hypotheses concerning protein function, albeit these two species diverged ~150M years ago and the extent of conservation of the *Hd1*-*Hd3a*/*CO*-*FT* modules is debated [32,33]. In Arabidopsis, CO can interact with several AtNF-YB and AtNF-YC subunits [34,35]. Induction of *FT* expression and flowering mediated by *CO* requires some of these subunits, because *nf-yb2 nf-yb3* or *nf-yc3 nf-yc4 nf-yc9* mutants fail to induce *FT* expression and flower late under LD [35,36]. Additionally, the early flowering phenotype of plants overexpressing *CO* from a strong promoter is limited by the double *nf-yb2 nf-yb3* or triple *nf-yc3 nf-yc4 nf-yc9* mutations [35,37]. The CO protein can directly bind the proximal promoter of *FT in vivo* [38] or *CO Response Elements* (*CORE*) *in vitro* [37]. Whether the recruitment of CO to the *FT* promoter is enhanced by the interaction with NF-YB and NF-YC subunits upon formation of a CO-containing NF-Y heterotrimeric complex is currently unknown. It is similarly unclear if rice relies on a trimeric NF-Y system to regulate expression of the florigens and flowering.

In this study, we used varieties flowering at higher latitudes to identify novel polymorphisms at loci relevant for photoperiodic adaptation. Two novel and common *Hd1* alleles were found, both sufficient to create a non-functional variant. Taking advantage of such genetic tools, we hypothesized and demonstrated the formation of a NF-Y heterotrimeric complex containing Hd1, capable of binding to a conserved response element in the *Hd3a* promoter. Genetic variation at *Hd1* can impinge on trimer formation and the floral transition. Our results suggest how multiple layers of variation can be stratified at the same locus and independently exploited during artificial selection. Additionally, they show how genetic diversity can provide unique molecular variants to understand specific developmental processes at the molecular level.

## Results

### The *Hd1*^*EH*^ allele promotes rice flowering under long days

**Fig 1:**
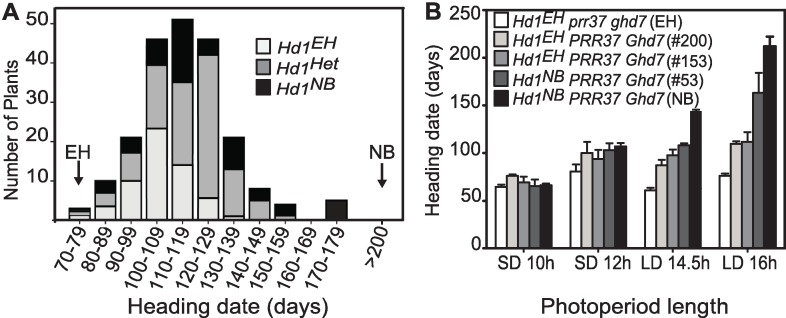
Identification of a novel *Hd1* allele conferring reduced sensitivity to photoperiod. **A**, normal distribution of heading dates of F2 recombinants derived from a cross between NB and EH and grown under LD (16L/8D). Distinct colors indicate the genotypes at the *Hd1* locus: light gray and black indicate the homozygous alleles of EH and NB, respectively, whereas dark gray indicates the heterozygous individuals. Arrows indicate the heading dates of the two parental lines. The *Hd1*^*EH*^ allele is associated with early heading individuals (t-Student’s test p<0.001). **B**, Heading dates of F3 lines containing the *Hd1*^*EH*^ and *Hd1*^*NB*^ allele compared to the parental genotypes and grown under several day length conditions. Two independent lines that harbor the *Hd1*^*EH*^ allele are shown. Numbers among brackets indicate the parental F2 plant.

Artificial selection of loss-of-function mutations in floral repressor genes, including *Hd1*, *Ghd7*, *Ghd8* and *PRR37* has been an effective strategy to expand rice cultivation to higher latitudes in both Asia and Europe [4,23,28,39,40]. However, the genetic determinants that allowed expansion have not been fully determined for all varieties and additional major regulators or novel haplotypes not previously described are likely to be present in several accessions. A genetic approach was used to identify candidate genes conferring reduced sensitivity to day length. A segregating population was obtained by crossing Nipponbare (NB) with Erythroceros Hokkaido (EH), a *temperate japonica* variety from Poland [29]. The genome of NB harbors functional *Hd1*, *Ghd7*, *Ghd8* and *PRR37* that confer sensitivity to day length (Photoperiod Sensitivity Index, PSI = 0.69), whereas EH is insensitive to the photoperiod (PSI = 0.16) and flowers very early regardless of external light conditions (Fig 1). Flowering of the resulting F2 individuals was scored under LD conditions (16L/8D) and followed a normal distribution (Fig 1A). The EH parental line was examined with molecular markers designed on known mutant alleles of floral repressors, revealing the presence of homozygous *ghd7-0a* and *prr37-2a* loss-of-function genes [14,16,23,40]. Consistently with this finding, the earliest flowering F2 plants co-segregated with these mutant alleles (S1 Fig). The *Hd1* locus of EH (*Hd1*^*EH*^) was sequenced, including the coding region (CDS), introns and untranslated 5’ and 3’ regions and was found to be identical to haplotype *Hd1*-VII [23]. The *Hd1*^*EH*^ allele apparently encodes for a functional protein, based on the absence of indels creating frame shifts or premature stop codons in the CDS. However, it co-segregated with very early heading plants, effectively behaving as a non-functional LD repressor or as constitutive activator of flowering (Fig 1A). To distinguish the effects of *ghd7*, *prr37* and *Hd1*^*EH*^ on flowering, F3 plants bearing single or multiple mutations were selected and heading dates were scored under natural long days (NLD) in Milan (45.47°N) (S2 Fig). Under NLD, the *Hd1*^*EH*^ allele strongly promoted flowering and pyramiding of *prr37* further accelerated it, indicating additive effects. Combinations of *prr37* and *ghd7* produced the shortest cycle length (S2 Fig). To assess the differences between *Hd1*^*NB*^ and *Hd1*^*EH*^ we selected F3 lines carrying wild type *Ghd7* and *PRR37* alleles and scored heading dates under increasing photoperiods (Fig 1B). Phenotypic differences between photoperiodic treatments were mild in lines harboring the *Hd1*^*EH*^ allele compared to lines harboring the *Hd1*^*NB*^ allele, confirming that genotypes containing *Hd1*^*EH*^ have reduced sensitivity to day length.

### Transcription of *Hd1* is suppressed in Erythroceros Hokkaido

**Fig 2:**
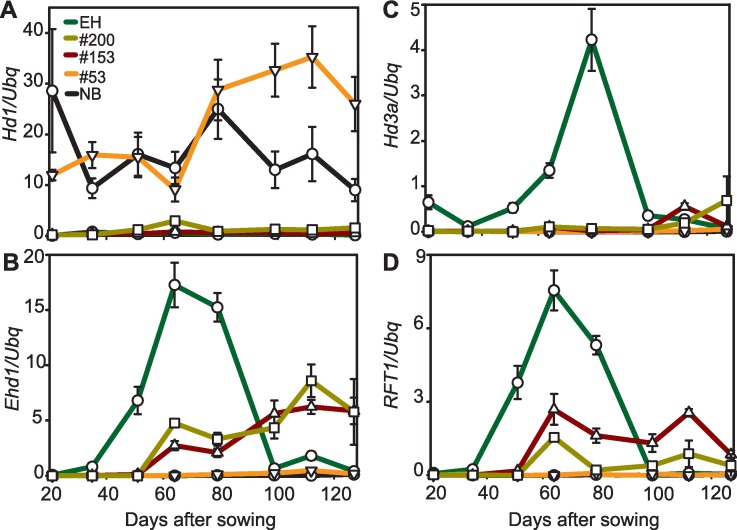
Expression of floral regulators is affected by the *Hd1*^*EH*^ allele. **B-E**, Quantification of mRNA expression of *Hd1* (**B**), *Ehd1* (**C**), *Hd3a* (**D**) and *RFT1* (**E**) from leaves of the indicated genotypes grown under LD (16L/8D). Transcription of *Hd1*^*EH*^ is extremely low during the entire time course. Error bars indicate the standard deviation.

To understand the functional effects of *Hd1*^*EH*^ on flowering, the mRNA levels of downstream targets of *Hd1* were quantified under LD conditions in selected F3 genotypes harboring the *Hd1*^*EH*^ or *Hd1*^*NB*^ alleles. The *Hd1*^*EH*^ allele caused precocious transcription of the florigens, and particularly that of *RFT1*, compared to plants carrying the *Hd1*^*NB*^ allele (Fig 2C and 2D). Expression of *Ehd1* was also elevated in plants bearing the *Hd1*^*EH*^ allele (Fig 2B). As *Hd1* represses *Ehd1* under LD conditions [23], increased transcription of *Ehd1* could be explained by reduced functionality or expression of *Hd1*. Interestingly, expression of *Hd1* from plants harboring the *Hd1*^*EH*^ allele was almost undetectable during the entire time course under LD (Fig 2A), a feature not previously described for any *Hd1* allele. Transcript abundance of *Hd1* shows a diurnal rhythm and to assess if low levels of *Hd1*^*EH*^ were caused by the time of sampling, diurnal time courses were collected and gene expression quantified during a 24h cycle under LD. Transcriptional levels of *Hd1* were strongly reduced in EH during the entire diurnal cycle, de-repressing *Ehd1* and promoting expression of the florigens (S3 Fig). Low levels of *Hd1* mRNA were detected also under SD, indicating that gene expression was not affected by the photoperiod (S3 Fig).

These data indicate that *Hd1*^*EH*^ is never expressed and it can be considered a loss-of-function allele. Silencing of *Hd1* is therefore an effective strategy to promote heading and indicates the existence of a tunable layer of variation creating phenotypic diversity.

### A mobile element is responsible for suppression of *Hd1* transcription in several varieties

**Fig 3:**
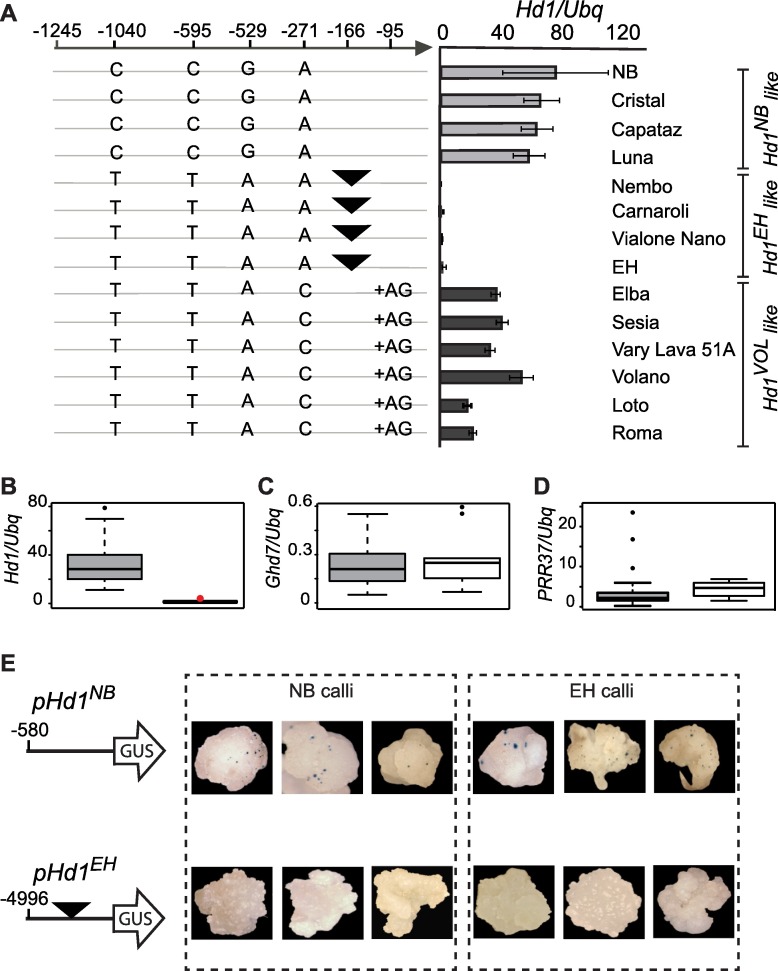
A mobile element suppresses transcription of *Hd1*. **A**, Comparison of the *Hd1* promoter haplotypes among 13 varieties belonging to the ERCC and Nipponbare. All varieties harbored functional alleles of *Ghd7*, *Ghd8* and *PRR37* (except EH). The position of each polymorphism is indicated relative to the ATG. The black triangle corresponds to the position of the mobile element (not drawn to scale). Varieties are clustered based on the presence of distinct alleles at the *Hd1* locus, including *Hd1*^*NB*^, *Hd1*^*EH*^ and *Hd1* from Volano (*Hd1*^*Vol*^), a widely cultivated, *temperate japonica* variety from Italy. Graphs on the right show *Hd1* expression under LD (16L/8D). Error bars indicate the standard deviation. **b-d**, Box plots showing expression levels of *Hd1* (**B**), *Ghd7* (**C**) and *PRR37* (**D**) quantified in 4-week-old seedlings of 102 accessions (“functional” varieties described in [23]) grown under 16h LD and distinguished based on the presence of the mobile element (gray: without mobile element, 38 varieties; white: with mobile element, 64 varieties). A single accession, Real, was heterozygous for the insertion of the mobile element (red dot). **E**, Expression assays in scutellum-derived calli transformed with an *Hd1*^*NB*^ or *Hd1*^*EH*^ promoter driving expression of a *GUS* reporter. Three representative calli are shown. For each condition, 20–25 calli were transformed and the experiment was repeated twice.

Regulatory elements in the *Hd1* promoter could be responsible for variation of its transcription. To test this hypothesis, fourteen varieties were chosen from the European Rice Core Collection (ERCC) that harbored functional alleles of *Ghd7*, *Ghd8* and *PRR37* and three distinct alleles at the *Hd1* locus, including *Hd1*^*NB*^, *Hd1*^*EH*^ and *Hd1* from Volano (*Hd1*^*Vol*^), a widely cultivated, high-yielding variety from Italy [41]. About 1.2 Kb of DNA upstream of the ATG was sequenced in this panel and all varieties carrying the *Hd1*^*EH*^ alleles were found to contain a sequence of ~4.4Kb at position -166bp, annotated as mobile element (GenBank accession AB300057.1) (Fig 3A). Whether this DNA sequence has features of a transposable or retrotransposable element is unclear. However, some sequences in the mobile element are expressed (Genbank accession AK101779.1). An additional copy of this same element is present in the rice genome, on chromosome 6.

*Hd1* was expressed in all varieties except those bearing the mobile element (Fig 3A). Using diagnostic primers, 242 varieties belonging to the ERCC were screened and 92 (38%) were identified that contained the mobile element (S1 Table). The same screen performed on a world panel, including 77 varieties belonging to all *Oryza* genetic groups, identified only two bearing the mobile element, suggesting that the non-expressed allele is more widely distributed among European varieties (S2 Table).

Among all varieties of the ERCC bearing functional copies of *Hd1*, *Ghd7*, *Ghd8* and *PRR37*, as defined by Gómez-Ariza *et al*. (102 accessions in total), two groups were distinguished based on the presence or absence of the mobile element (S1 Table). Expression of *Hd1* was quantified in 4-week-old seedlings grown under LD. Transcript levels were undetectable in varieties bearing the promoter insertion, whereas expression of *Ghd7* and *PRR37* was similar between the two groups (Fig 3B–3D). To clear the effects of the genetic background, we cloned the *Hd1* promoters of NB and EH, and used them to drive expression of the ß-glucuronidase gene (*GUS*) upon transformation into NB and EH calli. The *pHd1*^*NB*^*>>GUS* vector was active in both the NB and EH calli, whereas the *pHd1*^*EH*^*>>GUS* could not produce expression spots in EH and NB calli, indicating that failure to express the reporter was caused by *cis*-acting elements in *pHd1*^*EH*^ (Fig 3E). Taken together, these data indicate that insertion of a mobile element in the *Hd1* promoter prevents its expression. This is sufficient to reduce sensitivity to day length in several varieties and adapt them to European regions. These results also show how a transposable element has been instrumental to human selection to spread cultivation of a major cereal at higher latitudes, and add to the prominent roles that transposons have played during domestication and later diversification of crops [42–45].

### QTL-Seq identifies the *Hd1*^*Vol*^ allele and associates it with early heading under long days

**Fig 4:**
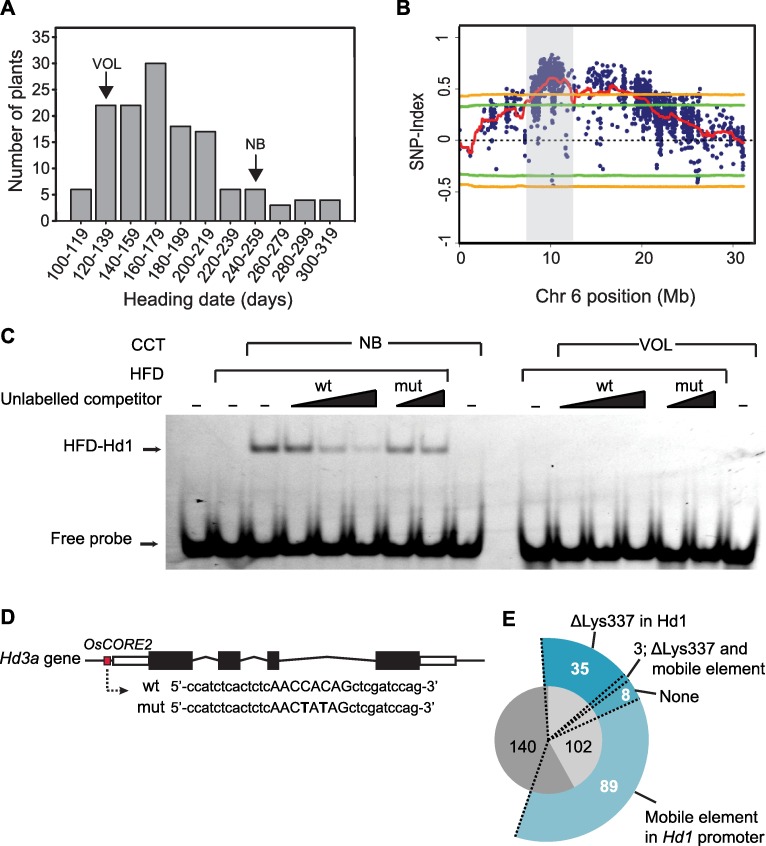
The OsNF-Y transcriptional repressor complex binds a response element in the *Hd3a* promoter. **A**, an F2 segregating progeny comprising 138 individuals from the VolxNB cross was grown under controlled LD of 16h light and heading dates showed a normal distribution. Heading dates of the parental genotypes are indicated by arrows. **B,** SNP-index plot showing a QTL on chromosome 6. The region highlighted spans the *Hd1* locus. Lines indicate statistical confidence intervals (green P<0.05, orange P<0.01). **C**, EMSA assays performed with distinct OsNF-Y complexes. A slow-migrating band is observed only when the heterotrimer is reconstituted using Ghd8 and OsNF-YC7 with Hd1^NB^ but not with Hd1^Vol^. Note that the intensity of the band does not decrease when an excess of mutated probe is incubated with the functional trimer. **D**, a map of the *Hd3a* locus showing the position of a *CORE2* site upstream of the 5’ UTR. White and black rectangles indicate the UTR regions and exons, respectively. Lines indicate the introns. The DNA probes used in EMSA assays are indicated, mut is the mutated probe harboring 2 mismatches in the *CORE* site (indicated by capital letters). **E**, summary of polymorphisms reducing sensitivity to day length in the ERCC. Dark gray indicates varieties bearing at least a non-functional *hd1*, *ghd7*, *ghd8*, or *prr37* gene; light gray corresponds to 102 varieties bearing functional coding sequences of floral repressors. The presence of varieties containing a mobile element in the *Hd1* proximal promoter, the ΔK337 polymorphism or both is indicated. Outer sectors indicate the number of varieties containing distinct allelic types. Eight varieties contain no polymorphism.

Of the 102 varieties belonging to the ERCC and categorized as having functional copies of LD floral repressors [23], 64 silence expression of *Hd1* through a mobile element inserted in its regulatory regions. From the remaining pool that expresses *Hd1*, Volano (Vol) was selected and crossed with NB to produce a recombinant population suitable for QTL mapping. An F2 segregating progeny comprising 138 individuals was grown under controlled LD (16L/8D) and heading dates were scored (Fig 4A). A normal distribution for days to heading was observed with several plants flowering very late and showing transgressive segregation. The DNA of twenty individuals from the earliest and latest flowering plants was bulked separately and a QTL-Seq approach was applied to identify the loci responsible for heading date variation [46]. A strong peak in the ΔSNP score was detected with high statistical significance on chromosome 6, representing the major locus controlling flowering in this cross (Fig 4B). The QTL corresponded to the position of the *Hd1* locus (Fig 4B). The normal distribution for days to heading suggested the existence of additional genes. Two QTLs were identified on chromosome 1 and 10 in which the NB allele promoted and delayed flowering, respectively. However, their statistical significance was lower compared to the QTL on chromosome 6, possibly due to low sequencing coverage and/or dominance effects of the QTLs (S4 Fig) [46].

The coding sequence of *Hd1*^*Vol*^ includes two in-frame insertions, several non-synonymous and conservative substitutions, and the deletion of a Lysine in the CCT domain, when compared to *Hd1*^*NB*^ (S5 Fig). Therefore, it is not interrupted by deletions or frame shifts and based on genetic evidences, it could be hypothesized that impaired function is caused by abolished mRNA expression, altered protein activity or mislocalization. Quantification of mRNA showed that *Hd1* transcripts abundance was reduced, but not abolished in Volano when compared to NB [23], and among the flowering repressors, also *Ghd7* and *PRR37* transcripts showed reduced diurnal cycling amplitude (S6 Fig). We transiently expressed the Hd1^NB^-GFP and Hd1^Vol^-GFP proteins in tobacco leaves under an inducible promoter and observed that both protein variants accumulated after induction and were targeted to the nucleus (S7 Fig). These data indicate that *Hd1*^*Vol*^ is expressed, cycles normally and is targeted correctly. We therefore tested whether the polymorphisms in Hd1^Vol^ prevent interactions with relevant partners binding to target genes.

### A heterotrimeric NF-Y transcription complex can accommodate Hd1^NB^ but not Hd1^Vol^

The AtNF-YB2/3 and AtNF-YC3/4/9 proteins are necessary for *FT* expression and flowering of Arabidopsis [34–36]. NF-YB and NF-YC form a histone fold domain (HFD) scaffold that accommodates NF-YA, the sequence-specific subunit of the *CCAAT*-binding trimer NF-Y [47–49]. Binding of AtNF-Y to the *CCAAT box* in the distal promoter of *FT* controls its expression and HFDs were shown to interact with CO [37,50,51]. In rice the corresponding components are encoded by *Hd1*, *Ghd8* (*OsNF-YB11*) and *NF-YC* subunits. Whether a NF-Y trimeric complex formed in rice and artificial selection of polymorphisms in single components affected features of the complex, including protein-protein interactions or DNA binding properties, has never been addressed. Assembly of a trimeric complex by distinct NF-Y components was therefore tested using yeast-two and three-hybrid assays. First, transcripts of the seven *NF-YC* genes encoded in the rice genome were quantified, to select for those expressed in leaves where the complex is likely formed. Transcripts of *NF-YC3* and *NF-YC5* were not expressed under LD conditions (S8 Fig); the remaining genes were all expressed at similar levels, except *NF-YC2*, whose diurnal oscillations were wider both under LD and SD (S8 Fig). The NF-YC1, NF-YC2 and NF-YC7 proteins were then expressed in yeast together with Ghd8. Heterodimeric interactions were observed for all combinations, as well as Ghd8 homodimerization (S3 Table). No direct interactions were detected between Hd1^NB^ or Hd1^Vol^ and NF-YC1, NF-YC7 or Ghd8. Therefore, we could not reproduce recent data indicating interaction between Hd1 and Ghd8, possibly because different cultivars were used [52]. As both BD:NF-YC2 and BD:Hd1 fusion proteins could autoactivate the yeast reporters, their interaction could not be determined.

The NF-YB and NF-YC subunits form the histone fold domain scaffold that accommodates the third subunit of the trimeric complex. Using yeast-three-hybrid assays, a strong interaction was observed between Hd1^NB^/NF-YC1/Ghd8 and Hd1^NB^/NF-YC7/Ghd8. However, Hd1^Vol^ could not interact with the NF-YC1/Ghd8 or NF-YC7/Ghd8 heterodimers (Table 1), indicating that some polymorphisms in Hd1^Vol^ prevent the formation of the heterotrimer.

**Table 1:** Formation of OsNF-Y heterotrimers between Ghd8, NF-YC and CCT proteins.

pGBKT7-Ghd8 ^a^		pGADT7			
		Hd1^NB^	Hd1^Vol^	PRR37^NB^	Empty AD
**pTFT1**	NF-YC1	+++ ^b^	-	+++	-
	NF-YC2	n.t.	n.t.	n.t.	++++
	NF-YC4	n.t.	n.t.	n.t.	++++
	NF-YC7	+++	-	+++	-
	Empty BD	-	-	-	-

^a^ The Ghd8 protein fused to the BD was expressed in yeast and used to bridge all interactions.

^b^ Interaction strengths are indicated by the capacity of yeast cells to grow on increasing concentrations of 3-amino-triazole (3AT). +++, 3AT 20mM; ++++, 3AT 30mM; -, no interaction on 3AT 30mM, n.t. not tested. The same interaction matrix generated using an empty pGBKT7 did not produce growth of yeast.

### OsPRR37 can form an alternative OsNF-Y heterotrimer

*OsPRR37* is a major LD repressor whose CCT-domain shows homology to the CCT of Hd1 and structural homology to NF-YA [16,40,53]. To address the combinatorial properties of the rice NF-Y complex, OsPRR37 was used in a yeast-three-hybrid assay together with NF-YC subunits and Ghd8. Growth of yeast on selective media indicated that the OsPRR37 protein could interact with the NF-YC1/Ghd8 and NF-YC7/Ghd8 heterodimers (Table 1).

Taken together, these data indicate that (i) a trimeric complex can be assembled between Hd1, Ghd8 and distinct NF-YC subunits, among which at least NF-YC1 and NF-YC7 interact strongly within the trimer, (ii) genetic variation creates protein variants unable to interact with the HFD and (iii) OsPRR37 (and possibly other PRR proteins) can replace Hd1 in the heterotrimer.

### Hd1^NB^ but not Hd1^Vol^ can bind a response element present in the promoter of *Hd3a*

The formation of a NF-Y trimer shown above and the resemblance of the CCT domain to the NF-YA domain required for *CCAAT*-binding [53], suggest that the CCT of Hd1 could impart sequence-specificity to the trimer, as well as being sufficient for heterotrimerization [34,50]. We assessed the DNA-binding properties of the complex by electrophoretic mobility shift assays (EMSA). We produced the HFDs of OsNF-YC7 and Ghd8, and the CCT domains of Hd1^NB^ and Hd1^Vol^ in *E*. *coli*. Note that CCT-Hd1^NB^ and CCT-Hd1^Vol^ differ only for a lysine, missing in Vol and lying in the first part of the CCT domain. This region, based on structural homology with the NF-YA A1 helix [53,54], is involved in protein-protein interactions with HFD proteins and highly conserved among CO-like proteins in monocots and dicots (S5 Fig). The DNA probe was selected within the *Hd3a* proximal promoter region, based on the presence and conservation of a *CO Response Element 2* (*CORE2*) located at -169bp [37,51]. Fig 4C shows that a shifted band was detected when CCT-Hd1^NB^/Ghd8/OsNF-YC7 were incubated together, reconstituting a trimeric complex. The band shift was not observed in the presence of CCT-Hd1^Vol^ or when Ghd8/OsNF-YC7 was missing (Fig 4C). To check for specificity, we challenged the complex with an unlabeled oligonucleotide identical to the DNA probe, or containing mutations in the *CORE2* site (Fig 4D). The wild type oligonucleotide, but not the mutated one, competed binding efficiently. Overall, these data corroborate the yeast analysis indicating that Hd1^NB^ forms a trimer with the OsNF-Y HFDs, and confirm that Hd1^Vol^, defective in HFDs association, is unable to bind a *CORE2* element in DNA-binding assays.

Finally, we checked the distribution of the Hd1^ΔK337^ polymorphism in the ERCC and found a total of 38 varieties sharing this mutation (Fig 4E). Notably, 33 of these belonged to the subset of 102 accessions mentioned above (S1 Table). Thus, the large majority of expressed variants of *Hd1* have been likely selected because they compromise the repressor function of the complex.

## Discussion

### An OsNF-Y protein complex links transcriptional regulators within the photoperiodic pathway

**Fig 5:**
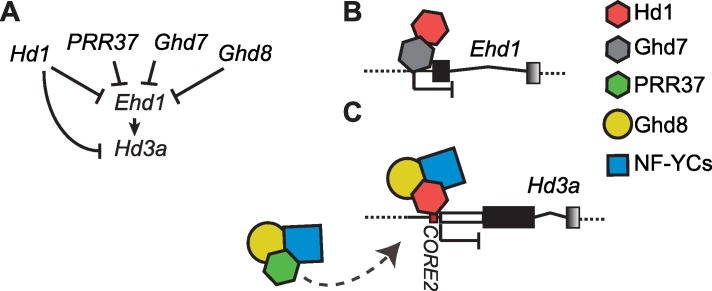
A model for the regulation of *Ehd1* and *Hd3a* expression. Schematic representation of the network repressing *Ehd1* and *Hd3a* expression (**A**). Flat-end arrows indicate transcriptional repression, whereas the arrow indicates transcriptional activation. Molecular view of repressor complexes bound to the *Ehd1* promoter (**B**), or to the *Hd3a* promoter (**C**). Hexagons indicate CCT domain proteins, circles and squares indicate NF-YB and NF-YC proteins, respectively. A PRR37-containing complex is indicated that could possibly compete with an Hd1-containing complex bound to *CORE2*.

The genetic architecture of the rice photoperiodic pathway heavily relies on floral repressor genes encoding transcription factors. These have been the first components to be isolated by genetic mapping in the flowering regulatory network and include *Hd1*, *Ghd7*, *Ghd8* and *PRR37* [14,16,25,55–57]. The position of such genes in the network initially suggested the existence of separate regulatory branches having partly unrelated effects. The *Ghd7* and *Ghd8* mutants have been isolated as independent regulators of *Ehd1* [15,55]. Mutations in *PRR37* have been initially believed to repress flowering by limiting *Hd3a* expression but not that of *Ehd1* or *RFT1* [16]. A later study indicated that *PRR37* acts upstream of both florigens by controlling *Ehd1* expression [40]. Until recently, the pathways centered on *Hd1* and *Ehd1* have been considered independent and acting in parallel, but recent data established a connection between Hd1 activity and *Ehd1* expression, demonstrating *Hd1* to be an upstream repressor of *Ehd1* under LD [23]. The data presented in this study indicate that Hd1, Ghd8 and PRR37 proteins do not act independently but rather assemble into a higher-order NF-Y protein complex that constitutes the molecular core of the photoperiodic pathway (Fig 5A). The recent demonstration of a molecular interaction between Hd1 and Ghd7 proteins at the *Ehd1* promoter, despite not directly implicating a heterotrimeric complex, further corroborates this interpretation [58]. Finally, binding of the heterotrimer to an element present in the *Hd3a* promoter suggests the existence of multiple targets for the OsNF-Y complex within the flowering network.

### Combinatorial properties of the OsNF-Y complex in the regulation of flowering time in rice

The NF-Y complex is a sequence-specific heterotrimeric transcription factor formed by histone-like subunits and common to eukaryotes [59]. However, whereas in animals and fungi each component of the complex is encoded by a single gene, plant genomes have largely amplified the number of subunits and in species such as rice or Arabidopsis hundreds of combinations of the NF-YA, B and C subunits are possible, that fine tune the spatio-temporal regulation of gene expression while enormously expanding the range of regulated processes [48,60–62].

In rice, the major NF-YB subunit involved in flowering time regulation is Ghd8 (OsNF-YB11), however, other components, including OsNF-YB10 and OsNF-YB8 share high sequence similarity with Ghd8 and might have a role as regulators of flowering in rice [53]. This view is corroborated by the fact that late flowering of Arabidopsis *nf-yb2 nf-yb3* double mutants is rescued by expression of *OsNF-YB10* or *OsNF-YB8*, suggesting an effect on flowering time control, at least in a heterologous system [63]. Additionally, overexpression of *OsNF-YB7* and *OsNF-YB9* delays flowering in rice under LD conditions [64]. Despite their sequence being only weakly related to that of Ghd8, they might compete with Ghd8 in the complex that includes it, or form an alternative one. Therefore, although Ghd8 is a prominent regulator, the existence of other NF-YB subunits regulating flowering in the Hd1/PRR37 containing complexes cannot be ruled out and deserves further attention.

Based on yeast interactions and DNA-protein binding assays we showed that OsNF-YC1 and OsNF-YC7 can interact with Ghd8 and CCT-domain proteins including, at least, Hd1 and PRR37. Trimer formation with OsNF-YC2 and OsNF-YC4 could not be formally demonstrated because of auto-activation of yeast reporters. However, dimeric interactions between OsNF-YC2 and OsNF-YB8, OsNF-YB10 and OsNF-YB11 have been reported [65]. Most importantly, genetic evidences support a role for OsNF-YC2 in the control of flowering in rice [65]. Transcriptional silencing of OsNF-YC2 by RNA-interference (RNAi) resulted in accelerated flowering under LD conditions, whereas its overexpression under the maize *ubiquitin* promoter strongly delayed flowering [65]. A milder effect on LD repression of flowering has been reported for OsNF-YC4, whereas OsNF-YC6 seems to have no role in flowering time control [65]. Taken together, these data indicate that in rice cells multiple HFD scaffolds can form and possibly bind to NF-YA or CCT domain proteins to control heading.

Demonstration of a heterotrimeric interaction between HFD dimers and PRR37 indicates for the first time that the HFD scaffold can bind proteins different from NF-YA or CO and CO-related proteins, all of which share a structurally similar, albeit not identical, CCT domain [34]. These findings further expand the combinatorial properties of the complex and might suggest a competitive mode of assembly, whereby Hd1 or other related proteins, including PRR-like or Ghd7-like factors, dynamically replace each other while interacting with the HFDs. Such model has been previously proposed for the CO2 and VRN2 proteins that were shown to compete with each other for binding to NF-YA, B or C components of wheat [47], but could be much more diversified among plant species as more CCT interactors become implicated in trimer formation. Additional combinations could be provided by direct interactions between CCT domain proteins. Recently, a direct interaction between Hd1 and Ghd7 was reported, and the Ghd7 protein was shown to bind the *Ehd1* promoter [58]. Whether Hd1 or other NF-Y subunits are required for Ghd7 binding to DNA is still unclear. Also, how the dynamical assembly of proteins around Hd1 is regulated is unknown. The NFYB/C dimers and Ghd7 could compete with each other for interacting with Hd1, similarly to CO2 and VRN2 wheat proteins, either at specific times of the day or season. Alternatively, Hd1 could be the scaffold on which both HFD proteins and Ghd7 interact, forming a large and unique LD repressor complex.

Since DNA specificity is determined by NF-YA or CCT domain proteins, a further layer of variation is provided by the sequences bound by such components, possibly being the *CCAAT* box [50], *CORE* elements ([37,51] and R.M., N.G. personal communication) or morning elements [66]. Finally, a crucial issue to address is when or in which cells an Hd1-containing complex is predominant over a PRR-containing complex to regulate expression of *Ehd1*, *Hd3a* or other genes, and how the dynamics of assembly and activity of alternative complexes are regulated at diurnal or seasonal levels. Not secondary to this question is the fact that since PRR proteins are central components of plants circadian clocks [67], the rhythm of expression of several genes other than those involved in flowering time control, might be dependent upon specific higher-order NF-Y complexes. Tissue-specific and temporal patterns of expression of *NF-Y* genes could help distinguishing between complexes possibly involved in regulation of photoperiodic responses (that take place in the vascular tissue of leaves or in cells of the shoot apex) from those involved in circadian clock functions (that take place in most cell types) [35,61].

### The photoperiodic pathways of rice and Arabidopsis share similarities and differences

Based on recent findings and on the results presented in this study, it is worth reconsidering the comparison between the photoperiodic regulatory networks of rice and Arabidopsis.

Day length responses in rice are not controlled by distinct pathways but by a unique one, whose regulatory elements converge on *Ehd1* [12]. Homologs of *Ehd1* have not been identified in Arabidopsis or other dicot species, but they are present in the genomes of monocots, thus encoding a function not shared by all plants, and that likely evolved after the split between monocots and dicots about 150M years ago [68,69]. The gene works as an upstream transcriptional activator of *Hd3a* and *RFT1* and promotes flowering under SD also in the absence of a functional *Hd1* [12,70]. However, its repression under LD is mediated by genes whose homologs are present in Arabidopsis, and function in the regulation of flowering time also in dicot species.

Similarly to Arabidopsis, the *OsGI*, *Hd1*, *Hd3a*/*RFT1* genetic cascade is present in rice as well.

The origin of the CO function and the conservation of the CO-FT module across Angiosperms has been challenged [32]. Simon et al., proposed that a CO function has evolved in the Brassicaceae family only after the most recent genome duplication that occurred within the family and that is not shared by its sister family [33]. Evolution of such function created a flexible switch to trigger flowering under LD. According to this interpretation, the Hd1 function might have evolved by convergent evolution. Consistent with a distinct origin (and distinct environmental pressures of tropical vs temperate areas), it is to be considered that a major function of Hd1 is to repress flowering under LD, and this function seems prominent compared to its function as SD flowering activator. These functions are not shared by Arabidopsis CO and the repressive activity in particular is directed to *Ehd1* [23,58]. Therefore, the Ehd1 function seems to have been added to, or co-evolved with an existing network containing homologs that are shared with Arabidopsis, and that *Ehd1* became central to the photoperiodic pathway of rice, as well as a hub gathering signals also from other environmental cues [71].

The DNA binding assays performed with heterotrimeric complexes indicates that the Hd1-containing NF-Y complex has the capacity to bind a *CORE2* element from the *Hd3a* promoter. Similar assays have demonstrated that CO can bind the *FT* promoter and that *CORE* sequences are necessary for binding [37]. Thus, protein-DNA interactions between Hd1-*Hd3a* and CO-*FT* suggest the existence of similar regulatory modules in rice and Arabidopsis. However, since the CO function evolved only recently in the Brassicaceae and the Hd1 function evolved by convergent evolution, the existence of such modules and their similar arrangement is striking [33]. This might be indicative of their robustness at the core of the photoperiodic pathway. The Hd1 protein could bind also to the *Ehd1* proximal promoter, as shown by chromatin immunoprecipitation assays, although no *CORE* sites have been identified in such region [58]. These data point to a three-node coherent feed forward loop of regulation under LD, directly linking Hd1 to *Ehd1* and *Hd3a* (Fig 5B and 5C). This mechanism might have evolved because the presence of an Ehd1 floral inductive function unlinked from an Hd1 repressive function could have resulted in the induction of *Hd3a*/*RFT1* expression also under LD. However, with both *Hd3a* and *Ehd1* under direct control of Hd1, this problem would be overcome and long photoperiods would prevent flowering by limiting all floral activators. The role of *RFT1* in such feed forward loop remains to be addressed. However, searches for *CORE* elements resulted in the identification of additional sites in both *Hd3a* and *RFT1* loci. Follow up studies *in vivo* will test if these can be effective binding sites for NF-Y repressor complexes.

## Methods

### Plant material and growth conditions

The Japanese rice variety Nipponbare (NB, *Hd1 Ghd7 PRR37 Ghd8 hd6*) was crossed with Erythroceros Hokkaido (EH, *Hd1*^*EH*^ *ghd7 prr37 Ghd8 Hd6*) to produce a recombinant F2 population comprising 215 individuals. Depending on the genotype, F2 individuals were selected and heading dates were determined using F3 plants under different photoperiodic conditions. Volano (Vol, *Hd1*^*Vol*^ *Ghd7 PRR37 Ghd8)*, a high-yielding variety from Italy, was crossed with NB and heading dates of 138 F2 individuals were scored.

The core collection comprising 242 varieties cultivated in Europe has been already described in[23]. Details of the accessions and genotypes of *Hd1*, *Ghd7*, *PRR37* and *Ghd8* are available in S1 Table.

Plants were grown under controlled conditions in Conviron PGR15 chambers or greenhouses under LD (16h light) or SD (10h light) photoperiodic regimes. Day and night temperatures were 28°C and 24°C, respectively. Humidity was set at 70% during the day and ~90% during the night.

Field experiments were performed at the Botanical Garden Città Studi, Milan (45.47°N). Seeds were sown in a cold greenhouse on Apr 11, 2014 and transplanted in an irrigated field on May 17, 2014. Heading dates were scored from ~30 plants/genotype. The photoperiod sensitivity index (PSI) was calculated as in [23].

### Preparation of genomic DNA, polymerase chain reaction and sequencing

Genomic DNA was prepared from leaves using a modified CTAB and chloroform:isoamyl alcohol method [72]. Genotyping of the NBxEH F2 population was performed using markers for the *prr37-2a* and *ghd7-0* alleles [23], whereas distribution of the *Hd1*^*NB*^ and *Hd1*^*EH*^ alleles was determined using primers listed in S4 Table. Genomic DNA was amplified using LA Taq from TaKaRa in Buffer I, according to manufacturer’s indications. For each PCR reaction, DNA was initially incubated five minutes at 95°C, followed by 40 cycles of amplification (95°C 30 seconds 58°C 30 seconds and 72°C 1 minute). The same PCR profile was applied to all PCR reactions, extending or shortening the extension time depending on the expected fragment size.

By using the same PCR conditions, the European rice varieties were screened for the presence of the 4.4Kb mobile element in the *Hd1* promoter, using forward 5’-promoter-anchored and reverse 3’-promoter-anchored primers in combination with primers designed within the mobile element. Additionally, a pair designed around the insertion site that could amplify only in the absence of the mobile element was used (S4 Table). Sequencing reactions were prepared and analyzed according to [23].

### RNA extraction and quantification of mRNA abundance

Total RNA was extracted using the TRI Reagent (Sigma Aldrich) from the distal part of young leaves collected from at least three independent plants. Genomic DNA was digested using TURBO DNAse (Life Technologies) and the RNA was precipitated with sodium acetate and ethanol and resuspended in water. After quantification of total RNA, 1μg was retrotranscribed with SuperScriptII Reverse Transcriptase (Invitrogen) and oligo-dT according to manufacturers’ instructions. The cDNA product was diluted 10 fold with sterile water. Transcripts were quantified in a Realplex^2^ (Eppendorf). Reactions were carried out using 3μl of cDNA as template, 5μl of 2X Maxima SYBR Green qPCR Master Mix (Thermo Scientific) and 0.2μl of each primer (final concentration 10μM) and ddH_2_O to a final volume of 10μL. A list of primers used for mRNA quantification is available in S4 Table. In particular, primers used to detect *Hd1* expression are located in the 3’UTR region, that was sequenced and found to be identical between *Hd1*^*EH*^ and *Hd1*^*NB*^, excluding the possibility that the primers used could not detect one of the two allelic variants.

### Transient expression in tobacco leaves

The coding sequence of *Hd1*^*NB*^ was fused at the C-terminus with mCherry and that of *Hd1*^*Vol*^ was fused with GFP in pABind vectors [73]. Expression of the fusion proteins was under a β-estradiol inducible promoter. Tobacco leaves were infiltrated with *Agrobacterium* cultures containing the plasmids. A 20μM β-estradiol solution was sprayed on leaves 3–12 hours before observation of epidermal cells using a confocal microscope.

### Yeast-two- and three-hybrid experiments

The coding sequences of genes used in yeast two- and three-hybrid assays were amplified from cDNA prepared from mature leaves using primers listed in S4 Table. The full length clone of *Ghd8* was synthesized by GENEWIZ Inc. (South Plainfield, NJ) whereas *OsPRR37* and NF-YC4 clones were obtained from the Rice Genome Resource Center (http://www.rgrc.dna.affrc.go.jp/index.html.en). All genes were cloned in pDONR207 (Life Technologies). Each entry clone was recombined with pGADT7 and pGBKT7 (Clontech), to obtain AD- and BD-fusion proteins.

The AH109 and Y187 strains were used in yeast transformation as described in the Clontech manual for the Matchmaker Gold yeast-two-hybrid system. Transformed cultures were selected on YSD media lacking leucine (Leu), tryptophan (Trp) or adenine (Ade) for pGADT7, pGBKT7 and pTFT1, respectively. Protein-protein interactions were assessed by streaking colonies on YSD media lacking Leu, Trp and Histidine (His) for Y2H experiments and on media lacking Leu, Trp, Ade and His for Y3H experiments. The strength of the interactions was evaluated by streaking colonies on increasing amounts of 3-aminotriazole (3AT). Yeast growth was verified after 6 days at 30°C. Each experiment has been repeated at least 3 times using independent clones.

### QTL mapping

The QTL-Seq approach has been previously described [46]. Briefly, DNA was prepared using the C-TAB method to extract genomic DNA individually from the twenty earliest and twenty latest flowering plants, within a total population of 138 F2s. DNA was quantified and two DNA pools of early and late flowering plants were produced using 1μg of genomic DNA per each plant. The whole genome was re-sequenced using Illumina HiSeq 2500 with chemistry v4 at Eurofins (Germany), producing 125bp paired ends reads. Whole-genome resequencing yielded 18896 and 24961Mbp, with an approximate coverage of 39 and 52 fold for the early and late flowering pools, respectively. Filtered short reads were aligned to the NB reference genome and SNP indexes were calculated for the early and late heading bulks. SNP values of less than 0.3 in both samples were removed and a ΔSNP-index was determined and plotted on the chromosome maps. Finally, using a sliding window analysis candidate QTLs were visualized.

### Protein purification

The cDNAs encoding the HFD of Ghd8 and OsNF-YC7 were synthesized by Eurofins Genomics and subcloned in pmcnCS EATCH using NdeI/BamHI restriction sites (S5 Fig). Only Ghd8 was tagged with 6x-His[74]. The CCT domains of Hd1^NB^ and Hd1^Vol^ were synthesized by Eurofins Genomics. The resulting proteins were tagged with 6x-His at the C-terminus and subcloned in pmcnEATCH. Soluble HFD heterodimers were produced in *E*. *coli* by co-expression in BL21(DE3) strains by IPTG induction, and purified using the HisSelect resin (SIGMA). The CCT domains of Hd1^NB^ and Hd1^Vol^ were purified separately. Proteins were eluted in Buffer A (10mM Tris pH8.0, 400mM NaCl, 2mM MgCl_2_), containing 250mM imidazole. Purified proteins were dialyzed against Buffer B (10mM Tris-Cl pH8.0, 400mM NaCl, 2mM DTT, 10% glycerol).

### Electrophoretic mobility shift assay

The protocol for EMSA was adapted from [48,50]. CCT-Hd1/Ghd8/OsNF-YC7 heterotrimeric complex assembly and *CORE2* DNA-binding was tested with Cy5-labeled oligos (Fig 4D). Ghd8/OsNF-YC7 dimers (60nM) and CCT-Hd1^NB^ or CCT-Hd1^Vol^ (360nM) were mixed in a final volume of 16μl with Cy5-5’-labeled *CORE2* probe in a reaction buffer (20nM ds-oligo, 12mM Tris-HCl pH8.0, 50mM KCl, 62.5mM NaCl, 0.5mM EDTA, 5mM MgCl_2_, 2.5mM DTT, 0.2mg/ml BSA, 5% glycerol, 100ng polydA-dT). To test the specificity of the binding, identical reactions were prepared with increasingly higher concentrations of unlabeled or mutated oligonucleotide competitors or TE buffer, as indicated in Fig 4C. Reactions were incubated at 30°C for 30min, and subsequently resolved by polyacrylamide gel electrophoresis. Fluorescence signals were detected using a Chemidoc MP system (Bio-Rad) with ImageLab software.

### Promoter analysis using GUS histochemical assays

The promoter regions of NB (580bp) and EH (4996bp including the mobile element) were amplified with primers suitable for Gateway cloning and recombined into pDONR207 vectors (Life Technologies). Positive clones were confirmed by sequencing and recombined into pBGWFS7, to drive expression of a GFP-GUS reporter gene, using LR clonase (Life Technologies).

Embryogenic calli of NB and EH were derived from scutella of mature seeds. For callus induction, seeds were dehusked, sterilized and placed on basal NB-medium plates (pH5.8), supplemented with 3mg/L 2,4D, 0.25mg/L cytochine and 30g/L glucose, for three weeks in the dark and 28°C. Proliferating embryogenic calli were subcultured on fresh medium for another three weeks before biolistic transformation.

Four hours before the bombardment and 16 hours after, calli were transferred on NB osmotic medium containing 34.6g/L of both mannitol and sorbitol, and they were kept in the dark at 28°C. After the osmotic treatment, calli were placed on basal MS medium supplemented with 3mg/L 2,4 D for two days before GUS staining. Non-bombarded calli and calli bombarded with gold microcarries only were used as negative controls.

The Biolistic PDS/1000 helium system (BioRad, USA) was used with the following parameters: ruptor disc pressure, 1100psi; macrocarrier to stop screening distance, 9cm; vacuum pressure, 28 inches of mercury (inHg); gold microparticle size, 1μm. For macrocarrier preparation, 4μg of plasmids were precipitated with 25μl of CaCl2 (2.5M), 10μl of spermidine (0,1M) and 25μl of gold particles (60mg/ml). The DNA bound to gold microcarriers were washed and re-suspended in ethanol. 10μl of gold microcarrier were then spotted on each macrocarrier for biolistic transformation. Biolistic transformation was repeated twice.

After bombardment, calli were incubated in the dark at 28°C for at least two days. The viable calli were then incubated in 90% cold acetone at -20°C for 20’, washed twice with NaPO_4_ buffer and finally transferred into the GUS histochemical reagent containing 1mM K_4_Fe(CN)_6_, 1mM K_3_Fe(CN)_6_, 0,1M NaPO_4_, 10mM EDTA, 0,1x Triton, 2mM X-Gluc. Samples were vacuum infiltrated for 20’ and then incubated for 24h at 37°C. After staining, samples were cleared with 70% ethanol.

## Supporting Information

S1 Fig The Ghd7 and PRR37 alleles from Erythroceros Hokkaido co-segregate with early heading genotypes grown under LD. **N**ormal distribution of heading dates of F2 recombinants derived from a cross between NB and EH and grown under LD (16L/8D). Distinct graphs indicate the genotypes at the *Ghd7* (**a**) and *PRR37* (**b**) loci. Light gray and black indicate the homozygous alleles of EH and NB, respectively, whereas dark gray indicates the heterozygous individuals. Arrows indicate the heading dates of the two parental lines. Mutations are associated with early flowering individuals (t-Student’s test p<0.0001 and p<0.005 for *PRR37* and *Ghd7*, respectively).
(EPS)

S2 Fig Effects of the combined EH alleles of Hd1, Ghd7 and PRR37 on flowering time. Heading dates of F3 families derived from the NBxEH segregating population and selected based on the genotypes of *Hd1*, *Ghd7* and *PRR37*. Plants were grown under NLD conditions in Milan (45.47°N) in 2014. Day length at this latitude is long and reaches 15h40m at the solstice, in the middle of the cropping season. Genotypes at the *Hd1*, *PRR37* and *Ghd7* loci are indicated on the x-axis for each F3 family, -/- and +/+ indicate homozygous EH or NB alleles, respectively. Numbers on the x-axis indicate the F2 individuals from which the F3s were derived. Two independent F3s are shown for each genotype, except for *Hd1*^*NB*^ *PRR37* -/- *Ghd7* +/+. Error bars indicate the standard deviation.
(EPS)

S3 Fig Expression of *Hd1* and downstream targets in NB and EH under long and short days. Diurnal expression of *Hd1* (**a**), *Hd3a* (**b**), *Ehd1* (**c**) and *RFT1* (**d**) in 8-week-old plants grown under LD (14.5L/9.5D). **e-h**, diurnal expression of *Hd1* (**e**), *Hd3a* (**f**), *Ehd1* (**g**) and *RFT1* (**h**) in 4-week-old plants grown under SD (10L/14D). Error bars indicate the standard deviation. White and black rectangles on top of the graphs indicate the duration of the day and night, respectively. ZT, *Zeitgeber*.
(EPS)

S4 Fig Identification of additional QTLs in the Volano x Nipponbare population. SNP-index plot represented for all chromosomes. Lines indicate statistical confidence intervals (green P<0.05, orange P<0.01). Note that additional QTLs are present in the middle of Chr01 and at the end of Chr10.
(EPS)

S5 Fig Comparison between Hd1 protein variants of NB and Vol, and clones used in Y3H and EMSA. **a**, comparison between the full length amino acid sequences of Hd1^NB^ and Hd1^Vol^. Substitutions in Hd1^Vol^ are indicated (note that numbers correspond to positions of the aminoacids in the NB sequence). The green rectangle indicates the B-BOXes and the orange rectangle the CCT domain. Insertions of 12 and 41 amino acids in Hd1^Vol^ are indicated with blue rectangles. **b**, structure of Ghd8 and NF-YC7, the yellow boxes indicate the two HFDs. Rectangles highlight the protein portion used in EMSA. **c**, details of the CCT domains used in EMSA to reconstitute a minimal OsNF-Y complex. The position of the only polymorphism distinguishing the NB and Vol CCT variants is indicated. The CCT domain shows structural similarity to the A1 and A2 helices of NF-YA. **d**, SDS-PAGE of proteins purified from *E*. *coli* and used in EMSA. For each sample, three different amounts (2, 4 and 8μg) were loaded. Dimers of OsNF-YC7 and 6xHis-Ghd8 were co-purified and appear as two bands. Relative mobility is consistent with the molecular weight of the fusion proteins.
(EPS)

S6 Fig Quantification of transcripts of floral repressors in Volano and NB. Quantification of *Hd1* (**a**), *PRR37* (**b**) and *Ghd7* (**c**) transcripts during diurnal time courses under LD (16L/8D). Six-week-old leaves were harvested from at least three independent individuals. Error bars indicate the standard deviation of three technical replicates. ZT, *Zeitgeber*.
(EPS)

S7 Fig Distinct Hd1 protein variants localized in the nucleus. Transient expression of the Hd1^NB^-mCherry (top) and Hd1^Vol^-GFP (bottom) proteins in tobacco epidermal cells upon induction with 20μM β-estradiol. Scale bar, 20μm.
(TIF)

S8 Fig Expression pattern of the OsNF-YC genes. Transcripts of OsNF-YC genes were quantified during diurnal time courses under LD (**a**) (14.5L/9.5D) and SD (**c**) (10L/14D) and during seasonal time courses under LD (**b**) (16L/8D) and SD (**d**) (12L/12D). Values represent the mean of at least three technical replicates. For seasonal time courses, leaf tissues were always collected at dawn (ZT0). Error bars indicate the standard deviation (±s.d.). Note that for OsNF-YC2 the normalized expression values are higher than that of other OsNF-YCs and they are plotted separately. White and black rectangles on top of the graphs indicate the duration of the day and night, respectively. ZT, *Zeitgeber*.
(EPS)

S1 Table Summary of genotypes of 242 varieties belonging to the ERCC collection. The table is a summary of the genotypes of the varieties used in this study, including the mobile element in the *Hd1* promoter and the ΔK337 polymorphism originally identified in Volano. For a complete overview of the genotypes, a summary of the data reported in Gomez-Ariza et al. is also shown on the right of the table. An X indicates the presence of a non-functional allelic variant. n.t., not tested. The 102 genotypes carrying functional LD floral inhibitors are indicated in yellow.
(XLSX)

S2 Table List of varieties belonging to the Rice Diversity Panel and including all rice genetic groups. Presence of the mobile element in the *Hd1* promoter is indicated by X. n.t., not tested.
(XLSX)

S3 Table HFD formation between Ghd8 and OsNF-YC subunits assessed by yeast-2-hybrid. Proteins were expressed in yeast as fusions to the GAL4 binding domain (in pGBKT7) or to the GAL4 activation domain (in pGADT7). Interactions were assessed on diploid yeast after mating between AD and BD clones, as represented. The strength of the interaction was quantified adding 3-amino-triazole (3AT) at increasing concentrations. A—sign indicates no interaction in plates that do not contain 3AT, +++ indicates strong interaction in plates containing 20mM 3AT. n.t., not tested. Interactions between two clones was not always tested upon swapping AD and BD domains.
(XLSX)

S4 Table List of primers used in this study. (XLSX)
