# Supplementary figures and images for: Transcriptional and Post-transcriptional Mechanisms Limit Heading Date 1 (Hd1) Function to Adapt Rice to High Latitudes
Source: PLoS Genet. 2017 Jan 9;13(1):e1006530. doi: 10.1371/journal.pgen.1006530 (PMC5221825; doi:10.1371/journal.pgen.1006530)

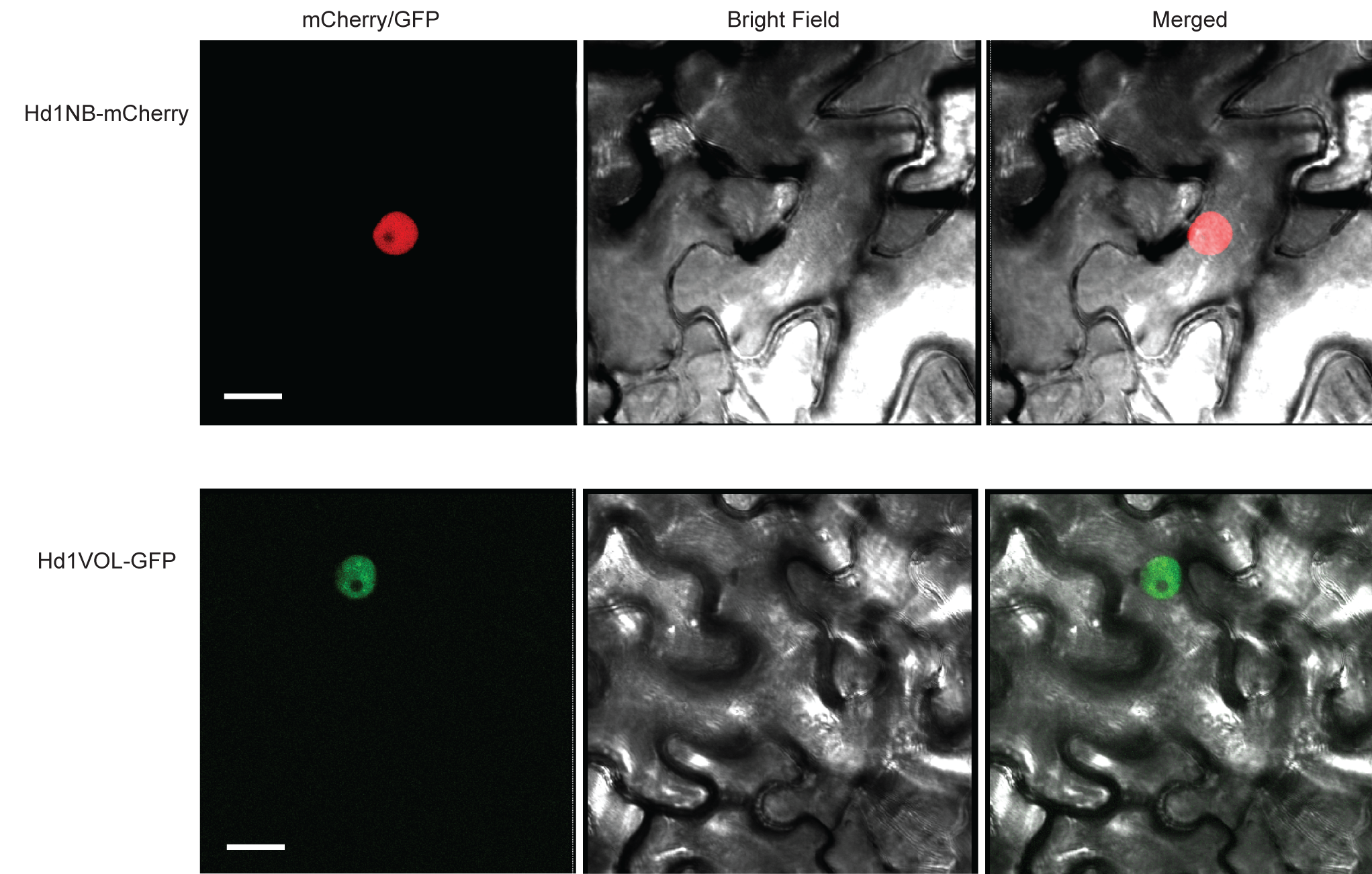

Supplement: S7 Fig — Transient expression of the Hd1NB-mCherry (top) and Hd1Vol-GFP (bottom) proteins in tobacco epidermal cells upon induction with 20μM β-estradiol. Scale bar, 20μm. (TIF) [file pgen.1006530.s007.tif]
